# Prospective associations between recalled parental bonding and perinatal depression: a cohort study in urban and rural Turkey

**DOI:** 10.1007/s00127-018-1484-3

**Published:** 2018-01-10

**Authors:** Berker Duman, Vesile Senturk Cankorur, Clare Taylor, Robert Stewart

**Affiliations:** 1 Department of Psychiatry, Ankara University Faculty of Medicine, Dikimevi, Ankara, 06100 Turkey; 2 King’s College London (Institute of Psychiatry, Psychology and Neuroscience), London, UK; 3 South London and Maudsley NHS Foundation Trust, London, UK

**Keywords:** Antenatal depression, Postnatal depression, Parental bonding, Parents, Cohort study

## Abstract

**Purpose:**

Recalled experiences of parental bonding may be important in the aetiology of perinatal depression. We hypothesized that lower recalled parental bonding would be associated with perinatal depression.

**Method:**

In a cohort study of perinatal depression in Turkey, 677 women were recruited in their third trimester. Parental Bonding Inventory (PBI) scores at baseline were investigated as predictors of depression on the Edinburgh Postnatal Depression Scale (EPDS) at 4, 14 and 21 months after childbirth in mothers without depression at baseline.

**Results:**

Poor parental bonding scores, apart from paternal control and overprotection, were independently associated with antenatal depression. Incident postnatal depression at 4 months was predicted by parental overprotection, at 14 months by parental care and overprotection, and at 21 months by paternal control and overprotection.

**Conclusions:**

Less satisfactory parenting recalled in the antenatal period was an independent predictor of postnatal depression; however, the different bonding subscales varied as predictors according to the timing of the depression assessment after childbirth.

## Introduction

Major depression is estimated to be the leading cause of disease-related disability among women in the world [1], who typically have a twofold increased risk of this disorder compared with men [2]. Depression occurring in the perinatal period is a particular concern, with a growing body of research into both antenatal and postanatal depression, encompassing depressive episodes that, respectively, occur during pregnancy or within the first 12 months after delivery [3]. However, estimates of prevalence for perinatal depression as a whole vary widely from 5% to more than 25% in pregnant women and new mothers [4].

The antenatal and postnatal periods can be viewed as a continuum, both with similar aetiologies. A number of risk factors for antenatal and postnatal depression have been identified, including low social support, a history of depression or depression during pregnancy, anxiety during pregnancy, low socioeconomic status and certain psychological characteristics such as low self-esteem [5]. In addition, poor relationships between women and their own parents have been reported as a risk factor [6], including parental rearing style and attachment [7, 8] and lack of care and overprotection in childhood more specifically [9–12]. Underlying mechanisms for these associations remain to be elucidated but the antenatal and postnatal periods may reactivate memories about original daughter–parent relationships, as well as being perceived as stressful periods in themselves. Prospective studies investigating perceived parental bonding as a risk factor for perinatal depression are relatively scarce. Therefore, in an analysis of a large cohort study of antenatal and postnatal depression carried out in Ankara, Turkey, we investigated the contemporaneous association between reported parental bonding skills and antenatal depression and the extent to which these predicted the incidence of postnatal depression.

## Method

### Study design, setting and recruitment sites

The source cohort study was carried out in and around Ankara, the capital of Turkey. The principal objective of this original study was to investigate factors associated with antenatal and postnatal depression in Turkish women, particularly focusing on social support from their husband, mother and mother-in-law, gender preferences, and the role of nuclear and traditional family structures in modifying these associations [13, 14]. The study described here was, therefore, a secondary analysis of pre-existing data. Baseline and first follow-up examinations have been previously described in detail [13, 14]. In summary, baseline samples were drawn from 20 urban and semi-rural antenatal clinics, where all women attending routine third trimester antenatal examinations were approached as participants between December 2007 and August 2008. Attempts were then made to re-contact and interview previous participants as close as possible to 2, 12, and 18 months after their childbirth. The study received approval from ethics committees at Ankara University Faculty of Medicine and King’s College London. After description of the study to the participants, written informed consent was obtained at all examinations.

## Measurements

### Socio-demographic information

Information was obtained at baseline on age, years of education, marital status, current physical health, previous mental health difficulties, life stressors, number of children, relationship quality with the husband and whether the pregnancy was planned or not. Self-reported general physical health was ascertained in three groups: very good, good, average and below. Previous mental health was categorised as a binary variable on the basis of any self-reported previous diagnosis of depression, other psychiatric illness or any past mental health problems. Participants were asked about the presence of the following life stressors/events within the last 12 months, and positive responses were summed and scaled [15]: being in debt, hunger from lack of food, recent separation, problems with friends, recent illness/injury, domestic violence, serious illness in a relative, death of a close family member, death of another relative, problems with a job, problems with money, problems with the justice system, and any robbery. Totalled numbers of recent life stressors were categorised into three groups as 0, 1, 2 and more. The relationship quality with the husband was assessed with a single question.

### Depressive symptoms

The Edinburgh Postnatal Depression Scale [16] (EPDS) was administered at all examinations. Although this has been principally applied to assess postnatal depression, it has also been used for antenatal depression. Also, the EPDS has found to have better screening properties than generic instruments such as the Beck Depression Inventory [17], is one of the most widely used screening instruments for perinatal depression internationally, and has been the most commonly used questionnaire for this purpose in Turkey. The EPDS maximum score is 30, and a score of 13 or above was used to classify case-level perinatal depressive symptoms (hereafter referred to as ‘depression’ for brevity), as has been most commonly applied in previous Turkish research [18]. The reliability and validity of the Turkish version has been previously established against the SCID as a gold standard, finding sensitivity and specificity of 0.76 and 0.71, respectively, and a Cronbach’s alpha value of 0.72 [18].

### Self-reported parental bonding

The Parental Bonding Inventory (PBI), administered at baseline, is a self-report questionnaire evaluating perceptions of how one was parented by recalling parents’ child-rearing attitudes before age 16 [19]. The scale consists of 25 items each for the father figure and mother figure separately, with recalled child-rearing attitudes evaluated on a four-point (0–3) scale for 12 care items, 7 overprotection items and 6 control items. Until now, there has been no clear consensus regarding the factor structure of the PBI. While some studies have reported a two-factor structure, other studies have suggested three- or four-factor solutions [20]. Psychometric properties of the Turkish version of the PBI have been evaluated in Turkish university students by Kapci and Kucuker [21], and the factor analysis for the Turkish version yielded two-factor solutions for both parents, as was the case for the original PBI; however, the items related to controlling behaviours are found to load on the care factor instead of the overprotection factor in the Turkish version [21]. Thus, these two factors are named as care/control and overprotection subscales. Because of this difference, rather than using derived factors as measures, we extracted raw scores for each subscale with respect to the mother and father and analysed them separately. These scores in which we used in the analysis were, therefore, as follows: maternal care (PBM care), maternal control (PBM control), maternal overprotection (PBM overprotection), paternal care (PBF care), paternal control (PBF control), and paternal overprotection (PBF overprotection). Higher scores indicate preferred parenting attitudes in all dimensions. The PBI was completed only once at the baseline assessment.

### Statistical analyses

The baseline sample was initially described with respect to the covariates and their associations with depression (EPDS caseness). PBI subscales were standardised by creating *z*-scores for each one to make interpretation and comparison easier. Associations of these standardised PBI subscales with depression caseness at baseline were analysed using logistic regression. Covariates were entered sequentially in the following groups: (1) Model 1 adjusting for age only; (2) Model 2 adjusting for age, number of children, duration of education; (3) Model 3 adjusting for age, number of children, duration of education, physical health and number of life stressors/events; (4) Model 4 adjusting for age, number of children, duration of education, physical health, number of life stressors/events and self-reported previous mental/emotional problems. For prospective analyses, we excluded depression cases at baseline and analysed identically defined depression at each follow-up examination as a binary variable, using identical logistic regression models to those at baseline. Further analyses accounting for potential dependency between repeated measurements of depression caseness were carried out using generalised estimating equation (GEE) binary logistic regression with an unstructured working correlation matrix. Covariates were entered as described for the logistic regression models. All statistical analyses were conducted using IBM SPSS Statistics Version 21.

## Results

Of 730 participants interviewed in their third trimester (95% of those approached for the study), 677 completed at least one subscale of the PBI and were included in the analysis presented here. Of these, 348 (51.4%) were reassessed at a mean (SD) 4.1 (3.3) months after childbirth, 294 (43.4%) at 13.7 (2.9) months and 280 (41.4%) at 20.8 (2.7) months. The main reasons for loss to follow-up were migration of families (16.9, 9.7 and 3.7%, respectively, for each follow-up) due to local re-allocation of housing around that time and consequent loss of contact; and refusal (5, 3.4 and 1.8% respectively). Attrition was not significantly associated with either depression or PBI scores at baseline (data not shown). Participants’ mean age at baseline was 26.1 years (SD 5.2, range 18–44), and their mean education duration was 8.4 years (SD 3.7). The majority (88%) reported a ‘good’ or ‘very good’ relationship with their husband, and in terms of the index pregnancy and childbirth, 79% reported that the pregnancy was planned; however, these were not included in analyses due to insufficient variance. Because almost all participants were married and cohabiting with their husband, this was not considered as a covariate. Nearly half of women (49%) had no children at the time of enrolment. Around a third of women (33.7%) had depression according to the EPDS ≥ 13 cut-off point at baseline. Unadjusted associations of covariates with depression are summarised in Table 1. Depression was associated with higher numbers of previous children, worse reported general health, recent life events/stressors, and self-reported past history of emotional problems. There were no significant associations with age or education level.

**Table 1:** Associations between participant characteristics and prevalence of case level depressive symptoms

	*n*	Depression prevalence (%)	OR	95% CI	*X*² (*df*)	*p* value
Age					6.29 (3)	*p* = 0.100
< 22	207	34.3	Reference			
23–25	165	37.6	1.15	0.75–1.77		
26–29	172	26.2	0.68	0.44–1.06		
> 30	168	36.9	1.12	0.73–1.71		
Child number					10.43 (2)	*p* = 0.005
0	376	31.9	Reference			
1	229	30.1	0.92	0.64–1.31		
≥ 2	111	46.8	1.88	1.22–2.89		
Education year					1.39 (3)	p = 0.707
≤ 5	232	33.2	Reference			
6–8	142	33.8	1.03	0.66–1.60		
9–11	242	35.1	1.09	0.75–1.59		
≥ 12	82	28.0	0.79	0.45–1.37		
Physical health					14.81 (2)	*p* = 0.001
Very good	129	31.0	Reference			
Good	443	30.2	0.97	0.63–1.48		
Average and below	141	47.5	2.02	1.22–3.32		
Life events					29.50 (2)	*p* = 0.000
0	378	25.1	Reference			
1	211	39.8	1.97	1.37–2.83		
2 and more	126	49.2	2.89	1.90–4.39		
Past mental health problems					59.85 (1)	*p* = 0.000
No	298	17.8	Reference			
Yes	402	45.8	3.90	2.73–5.57		

Associations between PBI measures and antenatal depression are summarised in Table 2. In unadjusted analyses, women with case level antenatal depression reported poorer parental attitudes in all dimensions of the PBI. Adjusting for covariates in general had negligible impact on the strength of the associations; however, coefficients for paternal control and overprotection scores fell below statistical significance levels in fully adjusted models.

**Table 2:** Association of self-recalled parental bonding with antenatal depression (*n* = 677)

PBI subscale	Unadjusted	Model 1	Model 2	Model 3	Model 4
Maternal care	**0.69 (0.59–0.81)**	**0.68 (0.58–0.80)**	**0.69 (0.58–0.81)**	**0.73 (0.61–0.87)**	**0.75 (0.63–0.90)**
Maternal control	**0.76 (0.64–0.89)**	**0.74 (0.63–0.88)**	**0.75 (0.63–0.89)**	**0.80 (0.67–0.96)**	**0.81 (0.68–0.98)**
Maternal overprotection	**0.69 (0.58–0.81)**	**0.67 (0.57–0.80)**	**0.68 (0.57–0.81)**	**0.67 (0.56–0.80)**	**0.69 (0.57–0.83)**
Paternal care	**0.76 (0.64–0.89)**	**0.75 (0.63–0.88)**	**0.74 (0.62–0.87)**	**0.76 (0.63–0.90)**	**0.81 (0.68–0.97)**
Paternal control	**0.81 (0.69–0.95)**	**0.80 (0.68–0.94)**	**0.81 (0.68–0.95)**	0.86 (0.72–1.02)	0.88 (0.74–1.06)
Paternal overprotection	**0.83 (0.70–0.97)**	**0.83 (0.71–0.98)**	**0.83 (0.70–0.98)**	**0.82 (0.69–0.98)**	0.84 (0.70–1.00)

Logistic regression coefficients represent odds ratios of depression for each standard deviation increment in subscale exposures

*Model 1* Adjusted for age

*Model 2* Adjusted for 1 and number of children, duration of education

*Model 3* Adjusted for 2 and physical health, number of life events/stressors

*Model 4* Adjusted for 3 and previous emotional problems

Significant values are in bold (*p* < 0.05)

Restricting the sample to those without case-level depression at baseline, incident postnatal depression at the first, second and third follow-up assessments was present in 13.7, 15.6 and 14.8%, respectively. Associations between baseline (antenatal) PBI subscale scores and incident postnatal depression outcomes are described in Tables 3, 4 and 5. In summary, four of the six subscales were significantly associated with postnatal depression at the second (14 month) follow-up, and associations with the remaining two subscales were close to statistical significance with coefficients largely unaltered following adjustment; however, associations were more limited at the first and third follow-up examinations: with maternal and paternal overprotection at the first (4 month) follow-up, and with paternal control and overprotection at the third (21 month) follow-up.

**Table 3:** Association between self-recalled parental bonding at baseline (third trimester) with incident depression at the first postnatal examination (*n* = 348)

PBI subscale	Unadjusted	Model 1	Model 2	Model 3	Model 4
Maternal care	0.96 (0.69–1.32)	0.98 (0.70–1.35)	0.99 (0.71–1.38)	1.02 (0.73–1.44)	1.04 (0.74–1.47)
Maternal control	0.94 (0.68–1.29)	0.94 (0.69–1.30)	1.00 (0.72–1.40)	1.02 (0.73–1.43)	1.03 (0.74–1.45)
Maternal overprotection	**0.70 (0.52–0.95)**	**0.66 (0.49–0.91)**	**0.68 (0.49–0.95)**	**0.68 (0.49–0.96)**	**0.70 (0.50–0.98)**
Paternal care	0.95 (0.67–1.33)	0.96 (0.68–1.35)	0.98 (0.69–1.38)	0.98 (0.69–1.40)	1.04 (0.72–1.49)
Paternal control	0.90 (0.65–1.27)	0.92 (0.65–1.30)	0.96 (0.67–1.36)	0.97 (0.67–1.4?)	1.03 (0.71–1.48)
Paternal overprotection	**0.66 (0.48–0.91)**	**0.63 (0.45–0.88)**	**0.63 (0.45–0.88)**	**0.63 (0.44–0.88)**	**0.64 (0.46–0.91)**

Mean (SD) 4.1 (3.3) months after childbirth

Logistic regression coefficients represent odds ratios of depression for each standard deviation increment in subscale exposures

*Model 1* Adjusted for age

*Model 2* Adjusted for 1 and number of children, duration of education

*Model 3* Adjusted for 2 and physical health, number of life events/stressors

*Model 4* Adjusted for 3 and previous emotional problems

Significant values are in bold (*p* < 0.05)

**Table 4:** Association between self-recalled parental bonding at baseline (third trimester) with incident depression at the second postnatal examination (*n* = 294)

PBI subscale	Unadjusted	Model 1	Model 2	Model 3	Model 4
Maternal care	**0.69 (0.51–0.95)**	**0.69 (0.50–0.95)**	**0.68 (0.49-0 0.95)**	**0.67 (0.48–0.94)**	**0.67 (0.47–0.94)**
Maternal control	**0.71 (0.52–0.97)**	**0.71 (0.52–0.97)**	0.73 (0.53–1.01)	0.74 (0.54–1.03)	0.74 (0.53–1.02)
Maternal overprotection	**0.56 (0.40–0.77)**	**0.55 (0.40–0.77)**	**0.51 (0.36–0.73)**	**0.51 (0.35–0.73)**	**0.50 (0.35–0.73)**
Paternal care	**0.67 (0.48–0.93)**	**0.67 (0.48–0.93)**	**0.67 (0.48–0.94)**	**0.67 (0.47–0.94)**	**0.64 (0.45–0.92)**
Paternal control	**0.70 (0.50–0.98)**	**0.69 (0.49–0.97)**	**0.70 (0.49–0.99)**	0.72 (0.50–1.03)	0.71 (0.49–1.03)
Paternal overprotection	**0.56 (0.40–0.79)**	**0.56 (0.40–0.79)**	**0.55 (0.39–0.77)**	**0.54 (0.38–0.77)**	**0.55 (0.39–0.78)**

Mean (SD) 13.7 (2.9) months after childbirth

Logistic regression coefficients represent odds ratios of depression for each standard deviation increment in subscale exposures

*Model 1* Adjusted for age

*Model 2* Adjusted for 1 and number of children, duration of education

*Model 3* Adjusted for 2 and physical health, number of life events/stressors

*Model 4* Adjusted for 3 and previous emotional problems

Significant values are in bold (*p* < 0.05)

**Table 5:** Association between self-recalled parental bonding at baseline (third trimester) with incident depression at the third postnatal examination (*n* = 280)

PBI subscale	Unadjusted	Model 1	Model 2	Model 3	Model 4
Maternal care	0.79 (0.57–1.10)	0.77 (0.56–1.08)	0.75 (0.53–1.07)	0.74 (0.52–1.06)	0.78 (0.55–1.10)
Maternal control	0.96 (0.69–1.34)	0.94 (0.67–1.32)	0.99 (0.70–1.41)	0.98 (0.69–1.39)	1.00 (0.70–1.44)
Maternal overprotection	0.75 (0.54–1.04)	0.75 (0.54–1.04)	0.78 (0.55–1.11)	0.80 (0.56–1.14)	0.81 (0.56–1.16)
Paternal care	0.75 (0.54–1.05)	0.74 (0.53–1.03)	0.75 (0.53–1.06)	**0.69 (0.48–0.98)**	0.73 (0.51–1.04)
Paternal control	**0.65 (0.46–0.93)**	**0.62 (0.43–0.89)**	**0.63 (0.43–0.93)**	**0.57 (0.38–0.86)**	**0.60 (0.40–0.91)**
Paternal overprotection	**0.62 (0.44–0.88)**	**0.62 (0.44–0.88)**	**0.63 (0.44–0.90)**	**0.65 (0.45–0.93)**	**0.63 (0.43–0.92)**

Mean (SD) 20.8 (2.7) months after childbirth

Logistic regression coefficients represent odds ratios of depression for each standard deviation increment in subscale exposures

*Model 1* Adjusted for age

*Model 2* Adjusted for 1 and number of children, duration of education

*Model 3* Adjusted for 2 and physical health, number of life events/stressors

*Model 4* Adjusted for 3 and previous emotional problems

Significant values are in bold (*p* < 0.05)

GEE binary logistic regression models are summarised in Table 6. Because effects of PBI subscales did not change over the repeated measures of depression caseness (no significant interactions between PBI subscale and time; data not shown), main effects are presented. In fully adjusted models, postnatal depression was significantly negatively associated with maternal care, paternal care and paternal overprotection.

**Table 6:** Association between self-recalled parental bonding at baseline (third trimester) with depression caseness at follow-up examinations (first, second and third postnatal examinations)

PBI subscale	Unadjusted	Model 1	Model 2	Model 3	Model 4
Maternal care	**0.66 (0.51–0.85)**	**0.65 (0.50–0.83)**	**0.65 (0.50–0.84)**	**0.65 (0.51–0.84)**	**0.66 (0.51–0.84)**
Maternal control	0.86 (0.70–1.05)	0.85 (0.70–1.04)	0.87 (0.70–1.06)	0.87 (0.69–1.10)	0.89 (0.71–1.11)
Maternal overprotection	0.92 (0.73–1.15)	0.91 (0.73–1.13)	0.93 (0.75–1.17)	0.95 (0.76–1.20)	0.96 (0.77–1.21)
Paternal care	1.17 (0.77–1.79)	**0.66 (0.53–0.82)**	**0.67 (0.53–0.84)**	**0.66 (0.52–0.83)**	**0.66 (0.52–0.84)**
Paternal control	**0.77 (0.60–0.99)**	**0.77 (0.59–0.98)**	**0.77 (0.60–1.00)**	**0.77 (0.59–0.99)**	0.79 (0.61–1.03)
Paternal overprotection	**0.73 (0.57–0.94)**	**0.72 (0.56–0.93)**	**0.73 (0.56–0.95)**	**0.74 (0.56–0.97)**	**0.76 (0.58–0.99)**

Generalised estimating equation (GEE) binary logistic regression coefficients represent odds ratios of depression for each standard deviation increment in subscale exposures

*Model 1* Adjusted for age

*Model 2* Adjusted for 1 and number of children, duration of education

*Model 3* Adjusted for 2 and physical health, number of life events/stressors

*Model 4* Adjusted for 3 and previous emotional problems

Significant values are in bold (*p* < 0.05)

## Discussion

Parental bonding has long been suspected to be an important exposure for mental health outcomes, enshrined as a consideration in clinical formulations, and it is reasonable to suppose that it may become particularly important in the perinatal period for women, although empirical research has been relatively sparse and limited internationally [22]. In a large cohort of Turkish women followed from the third trimester to over 20 weeks after childbirth, we found that reported poor parental bonding was associated with increased vulnerability for perinatal depression. Cross-sectional analyses indicated that all subscales of the PBI apart from paternal control and paternal overprotection were associated with antenatal depression. In mothers without antenatal depression, postnatal depression at 4 months was predicted by maternal and paternal overprotection scores, postnatal depression at 14 months by maternal and paternal care and overprotection scores, and postnatal depression at 21 months by paternal control and overprotection scores. All significant associations at all examination points were in the directions anticipated—i.e. higher quality parental bonding with lower risk of depression.

Most previous research has focused on cross-sectional associations between postnatal depression and parental bonding which is clearly potentially limited because of potential information bias—i.e. a woman’s mood state influencing her recollection of parental relationships and behaviour. For example, Hayakawa et al. [12], reported that perceived rearing as measured by PBI was not a strong risk factor for postpartum depression as measured by the EPDS, and that PBI scores were influenced by depressive symptoms at the time of interview, although Wilhelm et al. [23] on the other hand reported the relative stability of PBI over 20 years [12, 23]. To our knowledge, ours is the first cohort study which has investigated the association between perinatal depression and parental bonding attitudes with a relatively large sample size and prospective design. We were, therefore, able to minimise the influence of mood disorder, at least, at the time parental bonding was being retrospectively measured, and investigate the relationship with future rather than contemporaneous depressive symptoms.

There are some features of this study which require consideration when drawing inferences. Considering the outcome, ‘depression’ was defined as scores above an EPDS cut-off. Although EPDS has been widely used it is a screening instrument, it is not a tool for clinical diagnosis and cannot be assumed to generalise to depressive disorder as a diagnosis. The nature of the exposure also requires consideration. The factor analysis for Turkish version yielded two-factor solutions for both parents, as in original PBI; however, the items related to controlling behaviours are loaded on the care factor instead of overprotection in the Turkish version [21]. This, for example, does not allow a construct to be applied such as ‘optimal parenting’, which has been defined as ‘high care, low control’ in other studies, so we took a pragmatic decision to analyse unmodified subscale scores instead [7, 24]. Although the PBI is designed to identify key features of parental bonding, it is clearly a retrospective evaluation and is completed by the participant concerned, so cannot of course be assumed to be equivalent to parental bonding objectively measured in childhood. It is also conceivable that the scores on the different subscales might reflect wider aspects of the recalled childhood/family environment rather than those elements implied by each subscale title. Finally, it is possible that ratings were influenced by factors other than mood at the baseline examination and that there was unmeasured confounding—for example, no attempt was made to measure personality traits [7, 9].

Our findings for postnatal outcomes do support prospective associations and a role for parental bonding as an aetiological factor. This is consistent with previous research demonstrating that adverse early caring experiences are associated with vulnerability to perinatal depression in adulthood [24, 25]. However, the associations we observed were clearly not consistent across the three examinations and this requires consideration. Inconsistencies might reflect the number of analyses being carried out and represent type 1 statistical error. However, there appeared to be a sizeable divergence between associations which were present or not. For example, at the second (14 month) follow-up, four of the six scales were significantly and independently associated with the outcome, and associations with the remaining two scales were close to statistical significance with minimal evidence of confounding. On the other hand, at the first (4 month) follow-up, the two overprotection scales were significantly associated with depression and unaltered by adjustment, whereas the remaining coefficients were close to null values in all models. Only at the third (21 month) follow-up were coefficients more evenly distributed between null and statistically significant values. No coefficients at any examination were close to statistical significance in the opposite direction to that anticipated, which suggests that the hypothesised overall association (between less satisfactory bonding and increased risk of depression) was upheld. An alternative possibility is that the associations do genuinely vary with the timing of the depression. Also, previous stuides suggest that different risk factors may be implicated at in onset and persistence of depression [9, 25, 26]. Taking the third follow-up, the interval between the exposure and outcome might simply have obscured some of the associations; this assumes that PBI ratings in the antenatal period might have been influenced by parental relationships at that time. For example, paternal overprotection recalled in the antenatal period might represent a feature of family relationships which has a long-lasting presence and/or influence, accounting for its prediction of postnatal depression at all follow-up points. Recalled maternal overprotection, on the other hand, may only have an influence at earlier stages after childbirth, and might have diminished in influence by the third follow-up because of the participant achieving increasing independence as the child becomes older (particularly as the majority of the sample at recruitment were expecting their first child). The associations with recalled maternal and paternal care may exert most of their influences in the middle period of the follow-up, when the infant has begun to express personality but is still requiring high levels of physical care. However, these conclusions can only be viewed as tentative, since they are derived from exploratory analyses and require replication in independent samples.

When GEE analyses were carried out for repeated measures of depression caseness at follow-up examinations, these indicated that maternal care, and paternal care and overprotection scales were significantly negatively associated with postnatal depression. Also, no significant interaction between PBI subscales and time was observed. These findings partially overlap with logistic regression analyses results at different time points. The main difference observed was for the maternal overprotection subscale which was not found to be a predictor in GEE models. Multiple analyses mean that this should be interpreted cautiously, although it might reflect a strong contemporaneous association at baseline which accounts for all subsequent associations.

Our findings do at least indicate that associations between upbringing and psychopathology are likely to be complex issues. Long-term sequelae of early caretaking quality is a central tenet of attachment theory [27]. Insecure attachment can lead to anxiety over relationships and in turn difficulties in accessing social support thus increasing risk for depression [5]. Unhealthy child–parent relationships in early childhood may render women more prone subsequently to perinatal depression. Mothers with postnatal depression sometimes fail to bond with their infant, which has an adverse effect on the development of the newborn [28–31]. Screening during pregnancy using the PBI or equivalent scales would be worth considering for further evaluation, as it may provide a means to identify women at higher risk of postnatal depression, with the potential for psychological support as a preventative intervention. However, clearly the pathways underlying such associations and their timing require further investigation for such interventions to be developed and appropriately targeted.
